# Characterization of *Sarcoptes scabiei* cofilin gene and assessment of recombinant cofilin protein as an antigen in indirect-ELISA for diagnosis

**DOI:** 10.1186/s12879-016-1353-1

**Published:** 2016-01-22

**Authors:** Yu Zheng, Ran He, Manli He, Xiaobin Gu, Tao Wang, Weimin Lai, Xuerong Peng, Guangyou Yang

**Affiliations:** 1 Department of Parasitology, College of Veterinary Medicine, Sichuan Agricultural University, Wenjiang, China; 2 Department of Chemistry, College of Life and Basic Science, Sichuan Agricultural University, Wenjiang, China

**Keywords:** *Sarcoptes scabiei*, Cofilin, Immunohistochemistry, ELISA

## Abstract

**Background:**

Scabies impairs the health of humans and animals and causes heavy economic losses. Traditional diagnostic methods for scabies are inefficient and ineffective, and so far there is no commercial immunodiagnostic or molecular based test for scabies.

**Methods:**

Here, we used recombinant *Sarcoptes scabiei* cofilin protein as an antigen to establish indirect ELISA. *S. scabiei* cofilin is highly homologous to *Dermatophagoides farinae* Der f 31 allergen (90 % identity). The *S. scabiei* cofilin gene was cloned and expressed in *Escherichia coli* to obtain recombinant protein. Western blotting and fluorescence immunohistochemistry were carried out, and we established an indirect ELISA method and detected 33 serum samples from scabies infected rabbits and 30 serum samples from naïve rabbits.

**Results:**

Western blotting demonstrated that *S. scabiei* cofilin possessed good immunogenicity and fluorescence immunohistochemistry showed the *S. scabiei* cofilin is widespread in the splanchnic area of mites. In ELISA, a cut-off value of 0.188 was determined to judge experimental positive and negative serum values. Specificity and sensitivity of the ELISA were 87.9 and 83.33 %, respectively.

**Conclusions:**

Recombinant *S. scabiei* cofilin showed potential value as a diagnostic antigen. The ELISA method established could be used in clinical diagnosis and provide experimental information in minimal or asymptomatic infection.

## Background

*Sarcoptes scabiei*, as an ectoparasite, causes a disease named scabies which spreads worldwide in humans and companion animals, livestock and wildlife, such as dogs, sheep, goats, foxes, raccoons, camels, wombats, etc. Mites burrow into the stratum granulosum of the skin, feeding on epidermal cells and serum [1], and cause an intensely pruritic rash, which is usually more apparent at night [2]. Heavy scabiei named “crusted scabies” may immunocompromise patients, leading to severe damage of skin on a large scale or even death [3]. Scabiei can also facilitate secondary infection, resulting in a series of additional and serious complications, such as streptococcal infection, which can finally cause chronic kidney disease [4, 5]. Scabies impairs the quality of life in humans [6]. In animals, pruritus and hypersensitive dermatitis caused by mites may lead to economic loss because of depression in growth and feed conversion rates. Many countries and international organizations realize the importance of scabies, which is listed among the top 50 most prevalent diseases worldwide [7] and is treated as a “neglected tropical disease” [8].

Recently, an increasing number of *S. scabiei* antigens have been characterized and explored to reveal their role in mites and in the relationship between mites and hosts [9]. It is practical to produce *S. scabiei* recombinant proteins, which are useful tools to understand the biology of the mite and the immune responses of parasites and hosts. However, no commercial immunodiagnostic or molecular based tests are currently available for scabies.

Traditional diagnostic methods for scabies are normally based on clinical symptoms and on observed scratching of mites from patients and infected animals. Diagnosis depends on experienced physicians and the number of mites on infested hosts. It is hard to distinguish scabies from eczema, hairless tinea, lice and crab lice because of similar symptoms [10, 11]. As seroantibody is generated before the emergence of clinical symptoms, using *S.scabiei* antigens to detect antibodies in enzyme-linked immunosorbent assay (ELISA) is a promising diagnostic avenue. ELISA could be efficient and effective for the early stage diagnosis of infection and the examination of patients and infested animals [12].

Cofilin, a member of the actin depolymerizing factor (ADF) family, exists in nearly all types of eukaryotic cells and plays important roles [13]. Especially in the modulation of actin dynamics [14] and is involved with various cell activities because of the essential function of actin in many processes such as cell migration, morphogenesis, endocytosis and cytokinesis [15, 16]. There has been much research on the functions of cofilins in parasites. Tammana et al. [17] deleted the ADF/cofilin gene of *Leishmania*, which resulted in several aberrations in the process of cell division. Kumar et al. [18] found the overexpression of ADF/cofilin protein impaired flagellum assembly and consequently cell motility in *Leishmania donovani* [17, 18]. Makioka et al. [19] analyzed three ADF/cofilin family proteins of *Entamoeba invadens* in relation to encystation and excystation. Additionally, experiments on cofilin have been conducted in *Trypanosoma brucei*, *Eimeria tenella*, *Cryptocaryon irritans*, *Toxoplasma gondii* and *Plasmodium falciparum* [20–26].

This study reports the cloning, expression and fluorescent immunolocalization of *S. scabiei* cofilin and an assessment of recombinant cofilin protein for diagnosis of scabies by ELISA.

## Methods

### Source and sera

*Sarcoptes scabiei* maintained on rabbits in Parasite Laboratory of Sichuan Agricultural University were harvested and unfed before handling. Total RNA was extracted from mites using an RNA isolation kit (Waston, Shanghai, China) and transcribed into cDNA using a cDNA synthesis kit (Fermentas); cDNA was stored at −70 °C until assay.

Thirty-three blood samples were collected from rabbits infected with *S. scabiei* and 30 blood samples were collected from naïve rabbits to test cross-reactions, serum samples were also collected from rabbits infected with *Psoroptes cuniculi* and *Cysticercosis pisiformis*. All blood samples were stored at −20 °C.

New Zealand rabbits were used in this study and were handled in strict accordance with the animal protection laws of the People’s Republic of China (a draft of an animal protection law in China was released on September 18, 2009). All procedures were carried out in strict accordance with the Guide for the Care and Use of Laboratory Animals by the Animal Ethics Committee of Sichuan Agricultural University (Ya’an, China) (Approval No. 2011–028).

### Cloning of cofilin gene and sequence analysis

The cofilin gene of *S. scabiei*, identified from NCBI within an expressed sequence tag (EST) (GenBank: BG817660), was amplified with primers which were designed using Primer 5.0 Software (forward primer: 5ʹ-AAATGGCCTCAGGTGTAACT-3ʹ; reverse primer: 5ʹ-GGTGGGT2233GAGATAATTTAGTTTC-3ʹ) (Invitrogen, Beijing, China). The PCR cycling conditions were: 94 °C for 5 min, 30 cycles of amplification at 94 °C for 1 min, 56 °C for 1 min, and 72 °C for 1 min, followed by a final extension step at 72 °C for 30 min. After extraction and purification using a QIAquick Gel Extraction Kit, PCR products were cloned into vector pMD19-T (TaKaRa Bio Co. Ltd., Dalian, China). Then the plasmid was transformed into *Escherichia coli* strain DH5a (Fermentas) and sequenced by Invitrogen (Beijing, China). The amplified DNA sequence was analyzed and compared with the EST of *S. scabiei* cofilin using DNAMAN Software and compared against the NCBI database utilizing BLAST (http://www.ncbi.nlm.nih.gov/).

### Expression and purification of recombinant cofilin

*BamH*I and *Xhol* restriction enzymes were used to digest *S.scabiei* cofilin DNA fragments which were amplified by primers with the corresponding restriction sites (forward primer: 5ʹ-CGCGGATCCATGGCCTCAGGTGTAACT-3ʹ; reverse primer: 5ʹ-CCGCTCGAGGGTGAGATAATTTAGTTTC-3ʹ), using *S.scabiei* cDNA as template. The digested DNA fragments were then ligated into pET-32a (+) (Novagen, Germany) and transformed into *E. coli* DH5α cells. After certifying these clones were positive by colony-PCR, recombinant plasmids were extracted and transformed into *E. coli* BL21 (DE3) (Novagen) for protein expression. Subsequently, bacteria for expression were cultivated overnight in 1 L LB medium at 37 °C and then induced for 4 h using isopropyl-β-D-thiogalactopyranoside (IPTG) at a final concentration of 1 mM. The expression of recombinant cofilin was confirmed by SDS-PAGE and Ni-IDA Sefinose™ Resin (Bio-Rad) was used to purify the recombinant protein. The concentration of the purified protein was determined by biophotometer (Eppendorf).

### Western blotting

Recombinant cofilin protein was detected by SDS-PAGE and transferred to a nitrocellulose membrane for 1 h in an electrophoretic transfer cell (Bio-Rad, USA). At room temperature, the membrane was blocked in TBST (40 mM Tris–HCl, 0.5 M NaCl, 0.1 Tween 20, pH 7.4) in 5 % skimmed milk for 2 h and then incubated with rabbit antiserum (diluted 1:200 with 1 % skimmed milk-TBST) overnight. Next, the membrane was washed three times using TBST, each for 5 min, before being incubated with horseradish peroxidase (HRP)-conjugated goat anti-rabbit antibody (diluted 1:1000) for 1 h. The membrane was then washed as before, and protein signals were detected using diaminobenzidine (DAB) reagent (Tiangen, China).

### Fluorescence immunohistochemistry

In order to perform immunolocalization studies of *Sarcoptes scabiei*, antiserum against *S. scabiei* cofilin was raised in rabbits. Rabbit sera were collected before immunizing to provide reagents for negative controls. For the first immunization, 200 μg recombinant *S. scabiei* cofilin emulsified with equal volumes of Freund’s complete adjuvant (Sigma, USA) was injected subcutaneously. The second and third injection for boosting immunization were given mixing 100 μg protein with equal volumes of Freund’s incomplete adjuvant at a 2-week interval. Two weeks after the final injection, rabbit antisera were collected. The antibody titer was determined by ELISA. The immunoglobulin G (IgG) was further isolated from antisera using a Protein G- Sepharose column (Bio-Rad, USA).

Mites were fixed in 1%molten agarose and set in paraffin wax after solidification of the molten agarose, followed by using a rotary microtome to cut embedded mites into 5 μm thick sections. The sections were then rehydrated by immersing the slides successively in xylene twice for 10 min each, 100 % ethanol twice for 10 min each, 95 % ethanol for 5 min, 70 % ethanol for 5 min, 50 % ethanol for 5 min and lastly rinsing the slides with deionized H_2_O. Slides were treated to inactivate endogenous peroxidase by incubating in blocking buffer (3 % H_2_O_2_ in PBS) for 15 min at 37 °C and then immersed in 0.01 M sodium citrate buffer solution (pH 6.0) at 95 °C for 15 min for heat-induced epitope retrieval. After incubating in blocking buffer (5 % BSA in PBS) for 1 h at room temperature, tissue sections were then incubated with specific rabbit anti-cofilin antibodies (diluted 1:100 in TBS) overnight at 4 °C. After washing three times with TBS, the sections were then incubated with fluorescein isothiocyanate (FITC) goat anti-rabbit IgG (H+L) (AMRESCO, Texas, USA) (diluted 1:100) in 0.1 % Evans blue, at 37 °C, in the dark, for 1 h. From this step forward, samples were protected from light. Slides were mounted with an anti-fade mounting media and visualized using a fluorescence microscope.

### Set-up of indirect ELISA and detecting serum samples

After optimizing the conditions for ELISA, this procedure was followed: each well of a 96-well plate was coated with 100 μL purified recombination cofilin protein overnight at 4 °C. The protein was diluted in PBS-T (16 mM Na_2_HPO_4_, 5 mM NaH_2_PO_4_, 120 mM NaCl, 0.05 % Tween 20, pH 7.4) to a concentration of 5 μg/mL in advance. The next day, plates were washed with PBS-T three times, each wash for 5 min, and then incubated with blocking buffer (0.5) dried skimmed milk) at 37 °C for 2 h. From this step forward, plates were washed with PBS-T between incubations as described above. Serums to be tested in the ELISA were diluted 1:100 in PBS-T and 100 μL of the diluted serum solution were added to every well and incubated for 45 min at 37 °C. Then, the secondary antibody (goat anti-rabbit IgG-horseradish peroxidase (HRP) antibody) diluted 1:3000 in PBS-T was added and incubated for 1 h at 37 °C. To reveal the reaction, the substrate 3,3,5,5-tetramethylbenzidine (TMB) was added to every well (100 μL) and incubated for 15 min. Lastly, the same volume of 1 M H_2_SO_4_ was added to stop the coloration and plates were read in an ELISA-reader at an absorbance of 450 nm to determine the optical densities (OD).

In total, 33 serum samples from scabies infected rabbits and 30 negative controls from naïve rabbits were tested. Serum samples from rabbits infected with *P. cuniculi* (*n* = 6) or *C. pisiformis* (*n* = 6) were used as controls for the determination of cross-reactivity. Experimental controls including background (all antibodies replaced with PBS-T), omission of antigen, omission of primary antibody and omission of secondary antibody were included.

### Statistical analysis

As a measure of potential diagnostic performance, sensitivity and specificity were calculated: Sensitivity = ELISA positive samples/*S. scabiei* infected samples × 100 %; Specificity = ELISA negatives samples/*S. scabiei* non-infected samples × 100 % [27, 28]. The cut-off value between negative and positive results was calculated as the average measurement of the mange negative animals plus three times the standard deviation [29–31]. The coefficient of variation (CV) and the concordance correlation coefficient (CCC) were calculated by estimating the repeatability of the same samples measured within and between plates [32, 33].

## Results

### Recombinant cofilin and sequence analysis

**Fig. 1:**
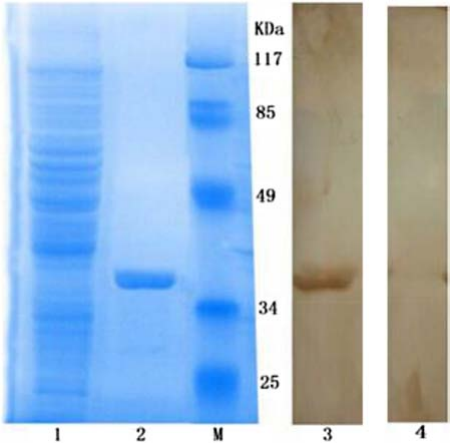
SDS-PAGE and Western blot of recombinant cofilin. Lanes: *M*: Protein molecular weight markers; *1*: Whole cell extracts from non-induced cells containing pET32a-cofilin; *2*: purified recombinant cofilin; *3*: Western blot detection with *S. scabiei* infected rabbit antisera *4*: Western blot detection with naïve serum

The clone of the *S. scabiei* cofilin gene was sequenced and compared against the NCBI database utilizing BLAST searching. The *S. scabiei* cofilin gene, consisting of 447 nucleotides, encoded 148 amino acids and was predicted to translate to a 16.8 kDa outer membrane protein. After optimizing the expression conditions, recombinant *S. scabiei* cofilin protein was produced by *E. coli* BL21 (DE3). Then a Ni-chelating column was used to purify the recombinant protein, which was examined by SDS-PAGE (Fig. 1).

### Western blotting and fluorescence immunohistochemistry

**Fig. 2:**
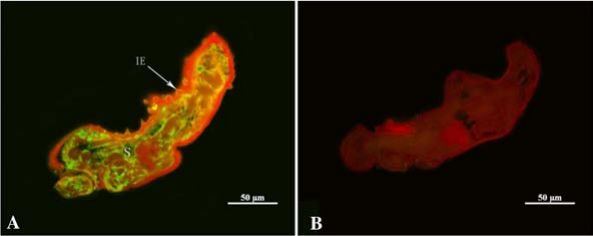
Immunolocalization of cofilin in sections of *Sarcoptes scabiei.* Panel **a**: staining with anti-cofilin as primary antibody; Panel **b**: control (no primary antibody). Annotation: S, splanchnic area; IE, epidermal integument

The recombinant protein reacted with sera from scabiei infested rabbits and there was no reaction between recombinant protein and negative sera (Fig. 1). Fluorescence immunohistochemistry showed *S. scabiei* cofilin is widespread in the splanchnic area of mites but not in the epidermal integument (Fig. 2).

### Indirect ELISA

**Fig. 3:**
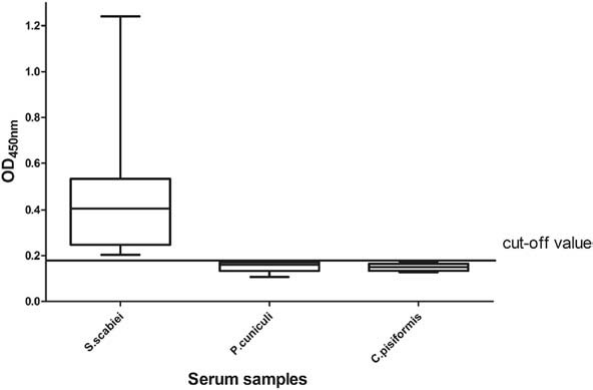
Cross-reactivity in ELISA between *S. scabiei*, *P. cuniculi* and *C. pisiformis. S.scabie*: OD_450_nm results of serums from *S.scabie* infected rabbits; *P. cuniculi*: OD_450_nm results of serums from *P. cuniculi* infected rabbits; *C. pisiformis*: OD_450_nm results of serums from *C. pisiformis* infected rabbits

**Fig. 4:**
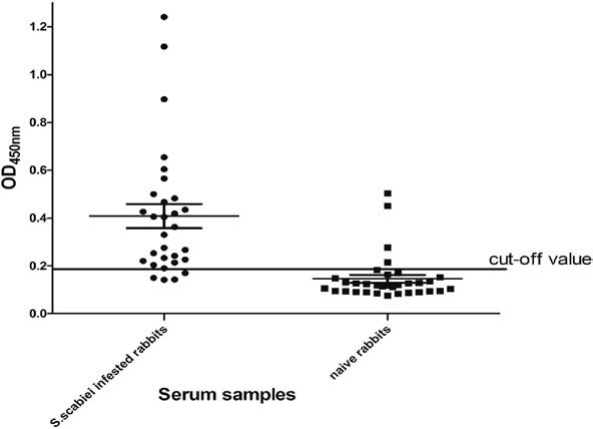
ELISA of serum samples from *S. scabiei* infected rabbits and naïve rabbits

The optimal coating concentration of antigen was 5 μg/mL, and the optimal working dilution of serum and secondary antibody was 1:100 and 1:3000, respectively. The cut-off value was 0.188; if OD_450_ ≥0.188, the serum was determined as positive, if OD_450_ <0.188, the serum was classed as negative. There was no significant cross-reactivity between the cofilin recombinant antigen and serum from rabbits infected with *P. cuniculi* or *C. pisiformis* (Fig. 3). The CV was 1.28–4.08 % and the CCC was 1.81–5.30 %. The sensitivity and specificity of the assay were calculated as 83.33 and 87.9 %, respectively (Fig. 4). Thus, recombinant *S. scabiei* cofilin protein has value as an antigen to be used in indirect ELISA for the diagnosis of scabiei.

## Discussion

This study characterized the *S. scabiei* cofilin gene and used its recombinant protein as an antigen in indirect ELISA for detecting the antibody in rabbit serum.

Western blotting showed the good immunogenicity of the *S. scabiei* cofilin protein and there was no band in negative controls. In fluorescence immunohistochemistry assay, *S. scabiei* cofilin located widely in the mites, which could be explained by the relationship between cofilin and actin.

Methods using recombinant allergens to diagnose parasite diseases such as infection with nematodes, cestodes, protozoa, hydatids and acaria are becoming prevalent [34–36]. Among these studies, recombinant antigens from mites such as thioredoxin peroxidase, Sar s 14.3, Pso o 2, Ssλ20Δ3 showed potential value for diagnosis. Sar s 14.3 and Pso o 2 are homologous with the allergens Der p 14 and Der f 2 of house dust mite, respectively [37–39]. Many allergens of house dust mite have been explored for diagnosis, of which Der p 1 is used for a reliable ELISA kit [40]. In our study, *S. scabiei* cofilin showed highly homology (90 % identity) with Der f 31, so it can be deduced that *S. scabiei* cofilin has potential value in exploring a standard procedure to diagnose scabies.

Although we observed no cross-reactivity between the recombinant cofilin antigen and the serum from rabbits infested with *P. cuniculi* or *C. pisiformis*, the OD_450nm_ of these samples were all close to the cut-off value. This phenomenon might result in error judgment for those data close to threshold, and illustrate cross-reactivity that cannot be clearly determined might exist; further study is need to solve this problem. CV (1.28–4.08 %) and CCC (1.81–5.30 %) values demonstrated that the operation of the ELISA was reliable.

We determined a cut-off value of 0.188 and calculated specificity of 87.9 % and sensitivity of 83.33 %. In recent research on diagnosis of *S. scabiei*, a study using recombinant Sar s 14.3 protein as antigen in dissociation-enhanced lanthanide fluorescent immunoassays (DELFIA) achieved 100 % sensitivity and 93.75 % specificity [37]. Kuhn immunoscreened six clones of sarcoptic mites to evaluate those expressed recombinant proteins as diagnostic antigens, in which the sensitivity ranged from 0 to 61.9 % [41]. The results of similar diagnostic research using recombinant proteins vary, probably because of the difference in the diagnostic assay applied in each study and/or the diversity in intrinsic characteristics of each antigen. According to Moendeg, cocktail-ELISA, mixing several good diagnostic antigens, might improve diagnostic potential and produce a method for multi-host species detection [42]. For scabies, further research is essential to establish a unified diagnostic method. Cofilin could be a potential diagnostic antigen for future diagnostic studies of scabies.

## Conclusions

In general, *S.scabiei* cofilin is widespread in the splanchnic area of *S. scabiei*. Recombinant *S. scabiei* cofilin showed potential value as a diagnostic antigen. *S.scabiei* cofilin based indirect ELISA for detection of scabies in animals is sensitive and specific, which means the *S.scabiei* cofilin could be used to develop an ELISA-based serological test for the diagnosis of scabies in animals.

## Abbreviations

ADF: actin depolymerizing factor
Der f: *dermatophagoides farina*
Der p: *dermatophagoides pteronyssinus*
*P. cuniculi*: *psoroptes cuniculi*
Pso o: *psoroptes ovis*
S.scabiei, Sar s, Ss: *sarcoptes scabiei*

## References

[1] Holt DC, Burgess STG (2013). Intestinal proteases of free-living and parasitic astigmatid mites. Cell Tissue Res.

[2] Currier RW, Walton SF, Currie BJ (2012). Scabies in animals and humans: history, evolutionary perspectives, and modern clinical management. Ann N Y Acad Sci.

[3] Karthikeyan K (2009). Crusted scabies. Indian J Dermatol Ve.

[4] Chung SD, Wang KH, Huang CC, Lin HC (2014). Scabies increased the risk of chronic kidney disease: a 5-year follow-up study. J Euracad Dermatol.

[5] Berríos X, Lagomarsino E, Solar E, Sandoval G, Guzmán B, Riedel I (2004). Post-streptococcal acute glomerulonephritis in Chile-20 years of experience. Pediatr Nephro.

[6] Worth C, Heukelbach J, Fengler G, Walter B, Liesenfeld O, Feldmeier H (2012). Impaired quality of life in adults and children with scabies from an impoverished community in Brazil. Int J Dermatol.

[7] Hay RJ, Johns NE, Williams HC, Bolliger IW, Dellavalle RP, Margolis DJ (2014). The Global burden of skin disease in 2010: an analysis of the prevalence and impact of skin conditions. J Invest Dermatol.

[8] Engelman D, Kiang K, Chosidow O, McCarthy J, Fuller C, Lammie P (2013). Toward the global control of human scabies: introducing the international alliance for the control of scabies. Plos Negl Trop Dis.

[9] Fischer K, Walton S (2014). Parasitic mites of medical and veterinary importance-is there a common research agenda?. Int J Parasitol.

[10] Walton SF, Currie BJ (2007). Problems in diagnosing scabies, a global disease in human and animal populations. Clin Microbiol Rev.

[11] Walton SF, Holt DC, Currie BJ, Kemp DJ (2004). Scabies: new future for a neglected disease. Adv Parasitol.

[12] Bornstein S, Zakrisson G, Thebo P (1995). Clinical picture and antibody response to experimental *Sarcoptes scabiei* var. vulpes infection in red foxes (Vulpes vulpes). Acta Vet Scand.

[13] Maciver SK, Hussey PJ (2002). The ADF/cofilin family: actin-remodeling proteins. Genome Biol.

[14] Bernstein BW, Bamburg JR (2010). ADF/cofilin: a functional node in cell biology. Trends Cell Biol.

[15] Poukkula M, Kremneva E, Serlachius M, Lappalainen P (2011). Actin-depolymerizing factor homology domain: a conserved fold performing diverse roles in cytoskeletal dynamics. Cytoskeleton (Hoboken).

[16] Bravo-Cordero JJ, Magalhaes MA, Eddy RJ, Hodgson L, Condeelis J (2013). Functions of cofilin in cell locomotion and invasion. Nat Rev Mol Cell Biol.

[17] Tammana TV, Sahasrabuddhe AA, Bajpai VK, Gupta CM (2010). ADF/cofilin-driven actin dynamics in early events of leishmania cell division. J Cell Sci.

[18] Kumar G, Srivastava R, Mitra K, Sahasrabuddhe AA, Gupta CM (2012). Overexpression of S4D mutant of leishmania donovani ADF/cofilin impairs flagellum assembly by affecting actin dynamics. Eukaryot Cell.

[19] Makioka A, Kumagai M, Hiranuka K, Kobayashi S, Takeuchi T (2011). Entamoeba invadens: identification of ADF/cofilin and their expression analysis in relation to encystation and excystation. Exp Parasitol.

[20] Dai K, Liao S, Zhang J, Zhang X, Tu X (2013). Structural and functional insight into ADF/cofilin from *Trypanosoma brucei*. PLoS One.

[21] Zhou BH, Wang HW, Xue FQ, Wang XY, Yang FK, Ban MM (2010). Actin-depolymerising factor of second-generation merozoite in Eimeria tenella: clone, prokaryotic expression and diclazuril-induced mRNA expression. Parasitol Res.

[22] Huang X, Xu Y, Guo G, Lin Q, Ye Z, Yuan L (2013). Molecular characterization of an actin depolymerizing factor from *Cryptocaryon irritans*. Parasitology.

[23] Allen ML, Dobrowolski JM, Muller H, Sibley LD, Mansour TE (1997). Cloning and characterization of actin depolymerizing factor from *Toxoplasma gondii*. Mol Biochem Parasitol.

[24] Ueno A, Dautu G, Saiki E, Haga K, Igarashi M (2010). *Toxoplasma gondii* deoxyribose phosphate aldolase-like protein (TgDPA) interacts with actin depolymerizing factor (TgADF) to enhance the actin filament dynamics in the bradyzoite stage. Mol Biochem Parasitol.

[25] Yadav R, Pathak PP, Shukla VK, Jain A, Srivastava S, Tripathi S (2011). Solution structure and dynamics of ADF from *Toxoplasma gondii*. J Struct Biol.

[26] Doi Y, Shinzawa N, Fukumoto S, Okano H, Kanuka H (2010). ADF2 is required for transformation of the ookinete and sporozoite in malaria parasite development. Biochem Biophys Res Commun.

[27] Rampton M, Walton SF, Holt DC, Pasay C, Kelly A, Currie BJ (2013). Antibody responses to *Sarcoptes scabiei* apolipoprotein in a porcine model: relevance to immunodiagnosis of recent infection. PLoS One.

[28] Yang D, Chen L, Wu X, Zhou X, Li M, Chen Z (2014). Expression of the Tpanxb1 gene from *Cysticercosis pisiformis* and its potential diagnostic value by dot-ELISA. J Parasitol.

[29] Sarkari B, Ashrafmansouri M, Hatam G, Habibi P, Abdolahi KS (2014). Performance of an ELISA and indirect immunofluorescence assay in serological diagnosis of zoonotic cutaneous leishmaniasis in iran. Interdiscip Perspect Infect Dis.

[30] Bornstein S, Wallgren P (1997). Serodiagnosis of sarcoptic mange in pigs. Vet Rec.

[31] Riyong D, Waikagul J, Panasoponkul C, Choochote W, Ito A, Dekumyoy P (2010). Size and charge antigens of *Dirofilaria immitis* adult worm for IgG-ELISA diagnosis of bancroftian filariasis. Southeast Asian Trop Med Public Health.

[32] Casais R, Goyena E, Martínez-Carrasco C, Ruiz de Ybáñez R, Alonso de Vega F, Ramis G (2013). Variable performance of a human derived Sarcoptes scabiei recombinant antigen ELISA in swine mange diagnosis. Vet Parasitol.

[33] Grabias B, Zheng H2, Mlambo G3, Tripathi AK4, Kumar S (2015). A sensitive enhanced chemiluminescent-ELISA for the detection of Plasmodium falciparum circumsporozoite antigen in midguts of Anopheles stephensi mosquitoes. J Microbiol Methods.

[34] Vlaminck J. Evaluation of *Ascaris suum* haemoglobin as a vaccine and diagnostic antigen. http://hdl.handle.net/1854/LU-3116855 (2013). Accessed 31 Jan 2013.

[35] Döşkaya M, Caner A, Can H, Gülçe İz S3, Gedik Y4, Döşkaya AD (2014). Diagnostic value of a rec-ELISA using *Toxoplasma gondii* recombinant SporoSAG, BAG1, and GRA1 proteins in murine models infected orally with tissue cysts and oocysts. PLoS One.

[36] Zheng W, Zhang R, Wu X, Ren Y, Nong X, Gu X (2014). Evaluating troponin C from *Psoroptes cuniculi* as a diagnostic antigen for a dot-ELISA assay to diagnose mite infestations in rabbits. Parasite Immunol.

[37] Jayaraj R, Hales B, Viberg L, Pizzuto S, Holt D, Rolland JM (2011). A diagnostic test for scabies: IgE specificity for a recombinant allergen of *Sarcoptes scabiei*. Eiagn Microbiol Infect Dis.

[38] Nunn FG, Burgess ST, Innocent G, Nisbet AJ, Bates P, Huntley JF (2011). Development of a serodiagnostic test for sheep scab using recombinant protein *Pso o* 2. Mol Cell Probes.

[39] Zhang R, Zheng W, Wu X, Jise Q, Ren Y, Nong X (2013). Characterisation and analysis of thioredoxin peroxidase as a potential antigen for the serodiagnosis of sarcoptic mange in rabbits by dot-ELISA. BMC Infect Dis.

[40] van Oeveren W, ten Brinke O, van der Graaf K (2011). Development of a enzyme-linked immunosorbent assay kit for determination of the major allergen from dermatophagoides pteronyssinus, Der p1, house dust mite. ALLERGY.

[41] Kuhn C, Lucius R, Matthes HF, Meusel G, Reich B, Kalinna BH (2008). Characterisation of recombinant immunoreactive antigens of the scab mite *Sarcoptes scabiei*. Vet Parasitol.

[42] Moendeg KJ, Angeles JM, Goto Y, Leonardo LR, Kirinoki M, Villacorte EA (2015). Development and optimization of cocktail-ELISA for a unified surveillance of zoonotic schistosomiasis in multiple host species. Parasitol Res.

